# Research on Adaptability Evaluation Method of Polymer by Nuclear Magnetic Resonance Technology

**DOI:** 10.3390/polym15040930

**Published:** 2023-02-13

**Authors:** Xiaocong Wang, Qun Lei, Jianhui Luo, Peiwen Xiao, Pingmei Wang, Yinzhu Ye, Sunan Cong, Xue Han, Lipeng He

**Affiliations:** 1 Research Institute of Petroleum Exploration & Development, PetroChina, Beijing 100083, China; 2 Key Laboratory of Nano Chemistry (KLNC), CNPC, Beijing 100083, China

**Keywords:** polymer flooding, nuclear magnetic resonance technique, molecular weight, swept volume, enhanced oil recovery

## Abstract

In order to study the matching relationship between polymer(HPAM) molecular weight and reservoir permeability, in this paper, the injection performance of polymers with different molecular weights in rock cores with different permeability is studied. Using nuclear magnetic resonance technology combined with conventional core displacement equipment, the change law of the displacement process was analyzed from three aspects of nuclear magnetic resonance *T_2_* spectrum, core layering, and imaging. Finally, the fluidity of the polymer solution in the core was analyzed by injection pressure control features. The experimental results show that the polymer solution with a molecular weight of 25 million has the best retention effect in the core flooding experiment and can stay in the dominant channel of the core for a long time to control the water flooding mobility. In rocks with a permeability of 500, 1000, and 2000 mD, subsequent water flooding can expand the swept volume by about 25% compared with polymer flooding. This method can effectively establish the adaptability matching relationship between the polymer molecular weight and the reservoir permeability.

## 1. Introduction

Polymers are used in a wide range between art, structure, outdoor equipment, oil, gas, aerospace, etc. [1,2,3]. Polymer flooding technology is one of the important technical means to enhance oil recovery in oilfields, and the effect of increasing oil is remarkable [4,5]. Laboratory studies and field tests have shown that polymer flooding can reduce the water/oil mobility ratio and expand the sweep coefficient of injected water in oil layers, and the viscoelasticity of polymers can improve microscopic oil washing efficiency [6,7,8]. The mechanism of enhanced oil recovery by polymer flooding is mainly to expand the swept volume.

The choice of polymer in oilfield applications needs to match the permeability of the reservoir [9]. If the hydration molecules of the polymer are relatively large, the phenomenon of polymer blockage in the formation is likely to occur during the migration of the polymer in the reservoir. The current method for evaluating polymer injectability is mainly to measure the size of polymer molecular coils. The measurement methods include numerical simulation, microporous membrane method, dynamic light scattering, microscope method, core flow experiment, etc. However, these methods require high measurement conditions, and the measurement results have certain limitations, so they are not suitable for situations where the number of samples to be tested is large [9,10,11].

Nuclear magnetic resonance (NMR) technology is widely used in the determination of fluid properties and rock properties and has the characteristics of speed, accuracy, and non-destructiveness [12,13,14]. Compared with traditional core displacement technology, NMR technology only collects the signal of fluid. By suppressing the fluid signal, different fluids in the core can be distinguished, and at the same time, quantitative measurement can be carried out, which is one of the important evaluation methods in the core displacement experiment.

In order to further tap the potential of polymers to enhance oil recovery, the main research object of this paper is the injection performance of polymers in reservoirs with different permeability. Firstly, nuclear magnetic resonance technology is used to monitor and analyze the change law of the core displacement process from three aspects of nuclear magnetic resonance *T_2_* spectrum, core layering, and imaging, and determine the main producing pore range of polymer flooding and water flooding. Then, through imaging and core layering, it is analyzed which part of the polymer plays a role in the core. Finally, compare the differences in injection pressure characteristics. The evaluation method of polymer adaptability is established to provide technical support for the promotion and application of polymer flooding technology.

## 2. Materials and Methods

### 2.1. Materials

Polymers (HPAM), molecular weight 16 million (solid content: 33.51%, viscosity: 6.19 mPa·s), molecular weight 19 million (solid content: 34.37%, viscosity: 6.96 mPa·s), molecular weight 25 million (solid content: 33.43%, viscosity: 7.91 mPa·s), provided by China Petroleum Exploration and Development Research Institute. Artificial cores (permeability 500 mD, 1000 mD, 2000 mD), purchased from Beijing Huarui Xincheng Technology Co., Ltd. (Beijing, China), The data are shown in Table 1. Simulated water (simulated Daqing Oilfield injection water, salinity 500 mg/L, prepared from pure water). Deuterium water, purity 99.9%, purchased from Iso-water Corporation (Ontario, Canada).

### 2.2. Experimental Equipment

**Figure 1:**
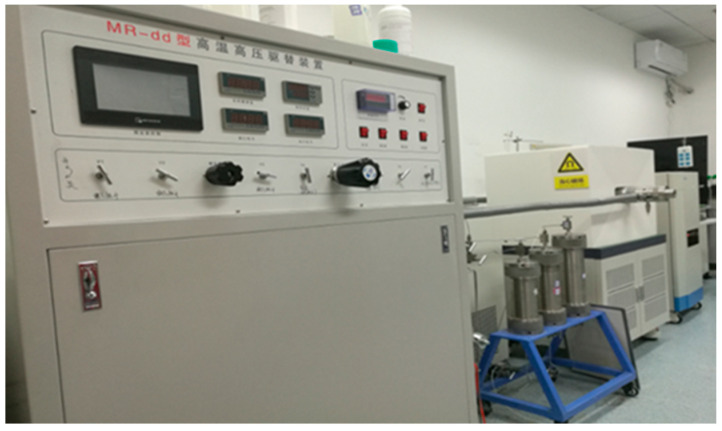
Diagram of low-field nuclear magnetic resonance displacement device.

**Figure 2:**
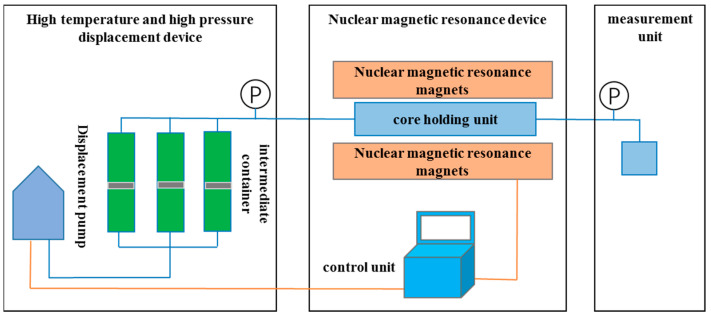
Schematic diagram of low-field nuclear magnetic resonance displacement device.

The NMR displacement device is mainly composed of a conventional displacement device and a nuclear magnetic resonance analyzer. The MR-dd high-temperature and high-pressure displacement device is produced by Nantong Huaxing Petroleum Instrument Co., Ltd. (Nantong, China). The MesoMR23-060H-HTHP core low-field nuclear magnetic analyzer is produced by Shanghai Numai Electronic Technology Co., Ltd. (Shanghai, China). The physical diagram and flow chart of the low-field NMR displacement device are shown in Figure 1 and Figure 2. As shown in the figure, the whole equipment is mainly composed of three parts: high temperature and pressure displacement device, nuclear magnetic resonance device, and metering device. The polymer and other fluids are placed in the intermediate container, the sample is placed in the core holder, and the fluid is injected into the core through the displacement pump. The nuclear magnetic equipment can be detected online, the experimental process does not need to be paused, and there is no need to repeatedly take out samples.

### 2.3. Experimental Principle

In the core displacement experiment, there are mainly two fluids in the core: water and oil (gas flooding is not considered). A large number of atomic nuclei ^1^H in water or oil fluid can reflect signals in nuclear magnetic resonance. However, there are pores of different sizes in the rock core, and the water or oil fluid in these pores, due to the change in the size or content of the space, the signals reflected in the nuclear magnetic resonance are also different. The detected signal can accurately reflect the distribution and change in the fluid in the core pores. [15].

**Figure 3:**
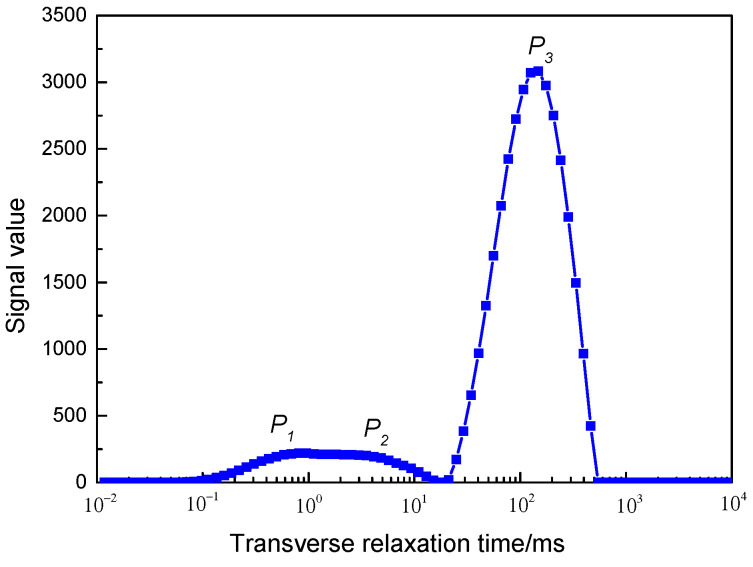
NMR *T_2_* spectrum of saturated water in high-permeability core.

The attenuation curve of the total NMR signal collected by the NMR test is multi-exponentially fitted to the echo string by using the Fourier mathematical inversion method [16,17]. The transverse relaxation time *T_2_* is used as the abscissa and the signal value as the ordinate, and the distribution of the *T_2_* time can be obtained, that is, the transverse relaxation *T_2_* spectrum curve (shown in Figure 3).

The obtained *T_2_* spectrum reflects the spatial distribution of the ^1^H proton-containing fluid in the core. The larger the transverse relaxation time, the larger the diameter of the pores where the ^1^H proton-containing fluid is located. Conversely, the smaller the transverse relaxation time, the smaller the diameter of the pores where the ^1^H proton fluid is located. The peak area enclosed by signal amplitude and relaxation time represents the total signal value of ^1^H proton fluid contained in the core pores, and the larger the peak area, the more ^1^H proton fluid is contained in the core pores. Conversely, less ^1^H proton fluid is contained in the pores [18,19]. Figure 3 shows the NMR *T_2_* spectrum curve of the core saturated with water. The area under the left peak (*P_1_* peak) represents the total signal value of the liquid in the small pores. The area of the middle peak (*P_2_* peak) represents the total signal of the liquid in the mesopores. The area of the right peak (*P_3_* peak) represents the total signal value of the liquid in the large pores. Therefore, during the core displacement experiment, by measuring the change in the transverse relaxation curve (*T_2_* spectrum) of the ^1^H-containing proton fluid in the core, the distribution of the ^1^H-containing fluid in different pores and the change in its content in the core can be obtained.

**Figure 4:**
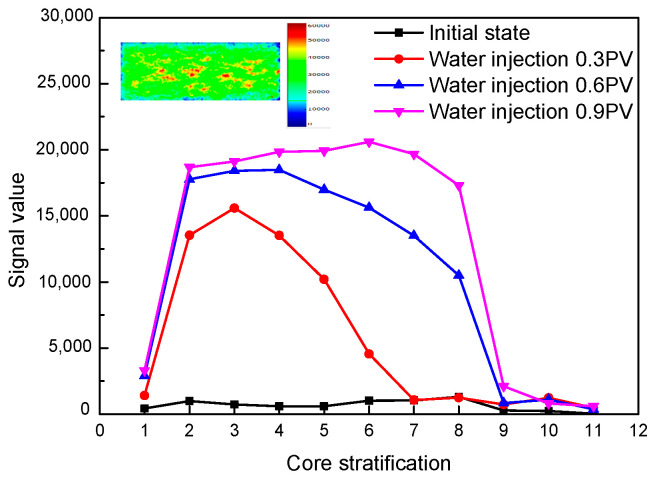
Variation trend of stratified nuclear magnetic signal during water flooding and NMR imaging after saturated water in high-permeability cores.

The layered sequence detection technology can divide the core into multiple sections for NMR signal monitoring. Figure 4 shows the signal change trend of each layer of the core and the nuclear magnetic imaging after water saturation during the water injection process of the high-permeability core. The length of the core sample is 5 cm, and the layered area is twice the length of the sample. As shown in the figure, the signal value is mainly reflected in the 2–8 layer, indicating that this area is the sample. The state of fluid migration in the core can be clearly reflected through layered sequence detection, and the distribution of fluid in the core can be directly observed by nuclear magnetic resonance imaging.

### 2.4. Experimental Method

In order to study the injection performance characteristics of polymers in cores, a method combining nuclear magnetic resonance technology and conventional core displacement equipment is used. First, select three polymers with different molecular weights (16 million molecular weight, 19 million molecular weight, and 25 million molecular weight), and prepare a 1000 mg/L polymer solution with simulated mineralized water. Secondly, three kinds of cores with different permeability (500 mD, 1000 mD, 2000 mD) were selected for core displacement experiments, and the displacement characteristics of polymer solutions with different molecular weights in cores with different seepage flow rates were compared and analyzed. All the core displacement experiments were carried out with a constant flow rate of 0.3 mL/min (converted into an actual reservoir flow velocity of 0.88 m/D, which is close to the flow velocity of the Daqing reservoir).

**Figure 5:**
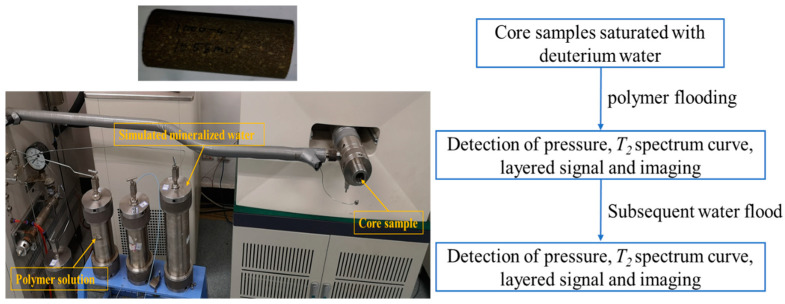
Experimental schematic diagram.

The specific experimental steps are as follows (As shown in Figure 5):

(1) The core is dried, and then vacuumed and saturated with deuterium water;
(2) Inject the prepared polymer solution at a constant flow of 0.3 mL/min until the injection pressure is constant, and obtain the NMR *T_2_* spectrum curve, layered signal, and imaging of polymer flooding;
(3) Continue to inject simulated mineralized water at a constant flow rate of 0.3 mL/min until the injection pressure is constant, and obtain the nuclear magnetic resonance *T_2_* spectrum curve, layered signal, and imaging of subsequent water flooding.

Main test parameters of nuclear magnetic resonance equipment: magnetic field strength 0.5 T, resonance frequency 21 MHz, probe coil diameter 70 mm; FID sequence parameters: receiver bandwidth SW 125 KHz, recovery time TW 3000 ms, number of sampling points TD 1024, RF coil dead time RFD 0.002, analog gain RG1 20 dB, digital gain DRG1 3, accumulation times NS 16; CPMG sequence parameters: receiver bandwidth SW 200 KHz, preamplifier position PRG 2, recovery time TW 3000 ms, echo time TE 0.3 ms, echo The quantity NE is 8000; the number of *T_2_* inversion fitting points is 100, and the minimum signal detection value is 5.23 × 10^−10^µg (water).

## 3. Results and Discussion

### 3.1. Variation Characteristics of Injection Pressure during Core Displacement

In order to ensure that the nuclear magnetic signal has no interference from external factors, in the experiment, the NMR signal detection of polymer flooding and subsequent water flooding was measured under the condition that the pressure at the inlet and outlet ports was atmospheric pressure. Therefore, the injection pressure changes during polymer flooding and subsequent water flooding were monitored from zero.

**Figure 6:**
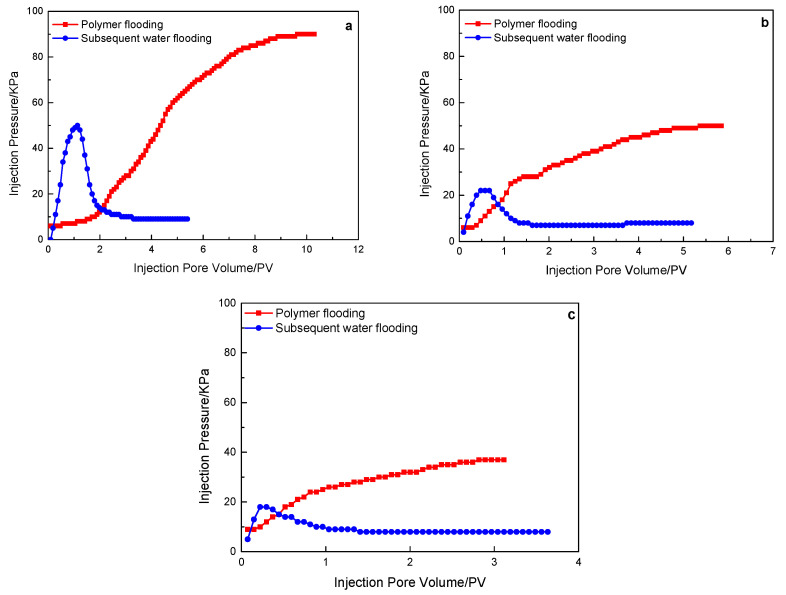
A 16 million molecular weight polymer solution in rock cores with different permeability, the characteristics of the injection pressure of polymer flooding and subsequent water flooding: (**a**) 500 mD core; (**b**) 1000 mD core; (**c**) 2000 mD core.

Figure 6 shows the injection pressure variation of the polymer solution with a molecular weight of 16 million in the flooding experiment of three different permeability rock cores. The red line in the three kinds of rocks is the pressure change curve of polymer injection, and the blue line is the pressure change curve of subsequent water flooding (the subsequent pressure curve color settings are the same). The final stable pressure of the polymer solution decreases with the increase in the permeability of the core, and the final stable pressure of the 500 mD, 1000 mD, and 2000 mD cores are 90 KPa, 50 KPa, and 37 KPa, respectively. In the subsequent water flooding process, the pressure first increased, and after 0.5 PV was injected, the pressure decreased, and after 1.0 PV was injected, the pressure began to stabilize, and the stable pressure was around 8 KPa. It shows that the polymer solution with a molecular weight of 16 million fails to stay in the core, further controls the mobility, and is displaced from the core with the increase in the subsequent water injection.

**Figure 7:**
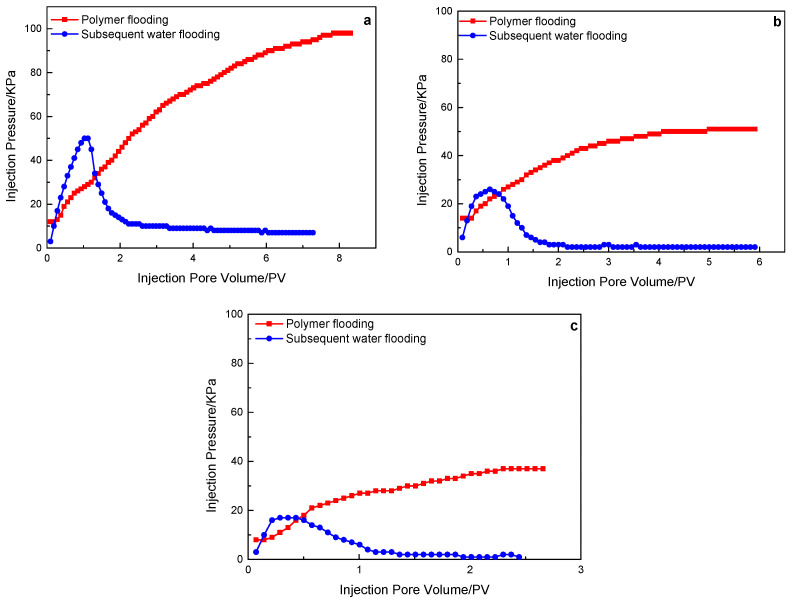
A 19 million molecular weight polymer solution in rock cores with different permeability, the characteristics of the injection pressure of polymer flooding, and subsequent water flooding: (**a**) 500 mD core; (**b**) 1000 mD core; (**c**) 2000 mD core.

Figure 7 shows the injection pressure variation of the polymer solution with a molecular weight of 19 million in the flooding experiment of three different permeability rock cores. Similar to the polymer solution with a molecular weight of 16 million, the polymer solution with a molecular weight of 19 million also failed to stay in the core and was displaced from the core with the increase in subsequent water injection.

**Figure 8:**
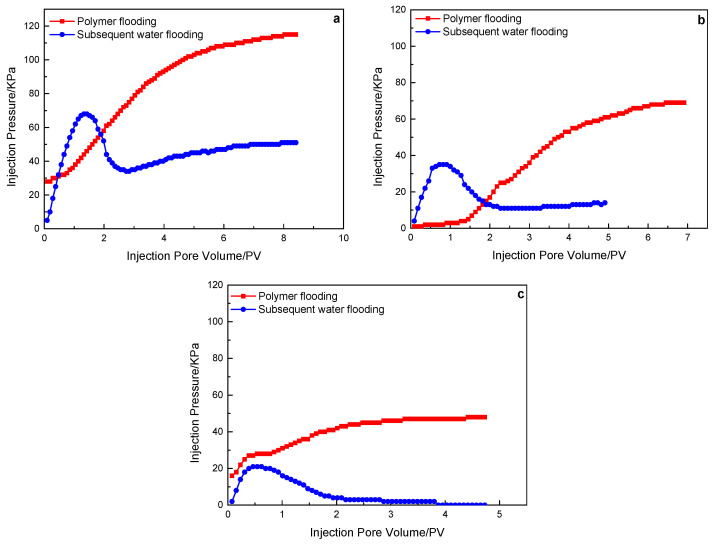
A 25 million molecular weight polymer solution in rock cores with different permeability, the characteristics of the injection pressure of polymer flooding, and subsequent water flooding: (**a**) 500 mD core; (**b**) 1000 mD core; (**c**) 2000 mD core.

Figure 8 shows the injection pressure variation of the polymer solution with a molecular weight of 25 million in the flooding experiment of rock cores with three different permeability. Different from polymer solutions with a molecular weight of 16 million and 19 million, polymer solutions with a molecular weight of 25 million can stay in 500 mD and 1000 mD cores for a long time, and the injection pressure can still be stabilized at 50 KPa and 11 KPa after water flooding for 5 PV. In the 2000 mD core, the polymer solution was gradually displaced after water injection of 2.0 PV.

To sum up, in the same permeability core, the injection pressure of a polymer with a molecular weight of 19 million is slightly higher than that of a polymer with a molecular weight of 16 million, while the injection pressure of a polymer with a molecular weight of 25 million is obviously higher than the former. It shows that the injection pressure increases with the increase in molecular weight. In the core flooding experiments of polymers with a molecular weight of 16 million and 19 million, the injection pressure of the subsequent water flooding decreased rapidly, indicating that it is difficult for such polymers to stay in the core for a long time. In the core flooding experiment of polymer with a molecular weight of 25 million, the subsequent water flooding declines slowly, and the final stable pressure is higher, indicating that this polymer can be better retained in the water flooding dominant channel of the core.

### 3.2. Change Characteristics of NMR T2 Spectrum in Core Displacement Experiments

**Figure 9:**
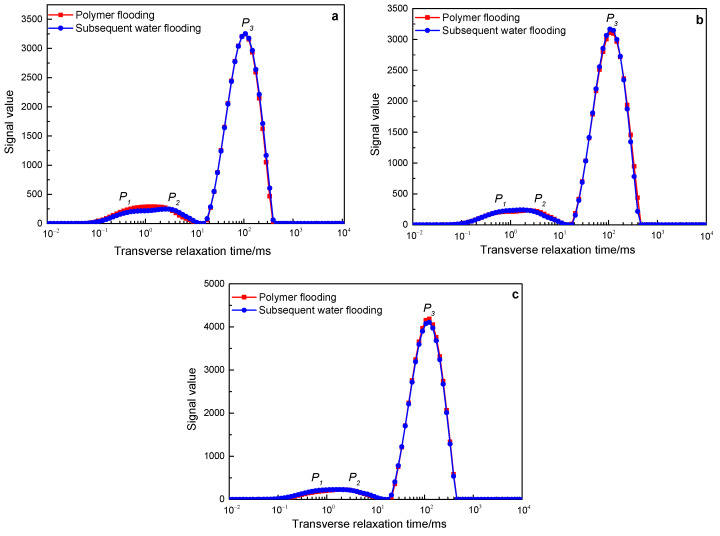
The change characteristics of the NMR *T_2_* spectrum curves of the polymer solution with a molecular weight of 16 million in rocks with different permeability after polymer flooding and subsequent water flooding: (**a**) 500 mD core; (**b**) 1000 mD core; (**c**) 2000 mD core.

Figure 9 shows the NMR *T_2_* spectrum change curves of polymers with a molecular weight of 16 million during the core flooding process of three different permeability. The red line is the NMR *T_2_* spectrum line measured after polymer injection, and the blue line is the NMR *T_2_* spectrum line measured after the subsequent water flooding (the color setting of the subsequent *T_2_* spectrum change curve is consistent with that in Figure 5). In the cores with three different permeabilities, the subsequent water flooding NMR *T_2_* spectrum curves coincide with the polymer flooding NMR curves, indicating that the subsequent water flooding failed to further expand the swept volume on the basis of polymer flooding.

**Figure 10:**
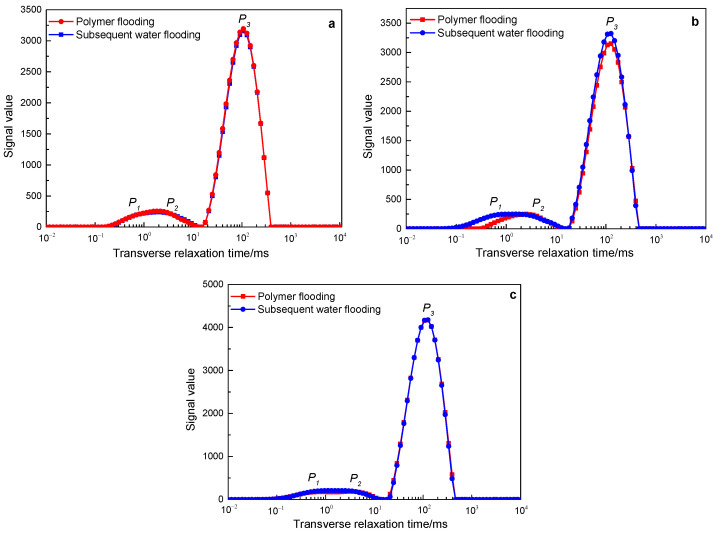
The change characteristics of the NMR *T_2_* spectrum curves of the polymer solution with a molecular weight of 19 million in rocks with different permeability after polymer flooding and subsequent water flooding: (**a**) 500 mD core; (**b**) 1000 mD core; (**c**) 2000 mD core.

Figure 10 shows the NMR *T_2_* spectrum change curves of polymers with a molecular weight of 19 million during the core flooding process of three different permeabilities. Among them, in the 500 mD and 2000 mD cores, the subsequent water flooding NMR *T_2_* spectrum curve coincides with the polymer flooding NMR curve, indicating that the subsequent water flooding failed to further expand the swept volume on the basis of polymer flooding. In the 1000 mD core, the signal of the follow-up water flooding has improved on the basis of polymer flooding, mainly reflected in the P1 peak (small pores) and *P_3_* peak (large pores). The total nuclear magnetic signal value of polymer flooding is 32,851, and the total nuclear magnetic signal value of subsequent water flooding is 36,377. According to the calculation according to Formula (1), subsequent water flooding expands the swept volume by 10.07% on the basis of polymer flooding.

(1) $$I=\frac{S_{W}-S_{P}}{S_{P}}\times 100\%$$

*I*—Sweep volume increase rate, %; *S*_P_, *S*_W_—NMR *T_2_* spectrum peak area of polymer flooding and subsequent water flooding, dimensionless.

**Figure 11:**
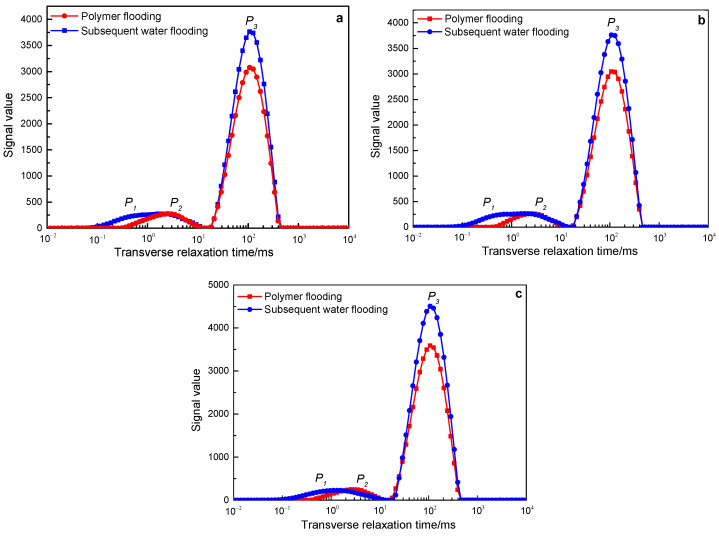
The change characteristics of the NMR *T_2_* spectrum curves of the polymer solution with a molecular weight of 25 million in rocks with different permeability after polymer flooding and subsequent water flooding: (**a**) 500 mD core; (**b**) 1000 mD core; (**c**) 2000 mD core.

Figure 11 shows the NMR *T_2_* spectrum change curves of polymers with a molecular weight of 25 million during the core flooding process of three different permeability. In the three core flooding experiments with different permeability, the subsequent water flooding increased the signal value on the basis of polymer flooding, and it was mainly manifested in the P3 peak (large pores), and the signal of the P1 peak (small pores) increased slightly. It shows that subsequent water flooding further expands the swept volume on the basis of polymer flooding. Calculated according to Formula (1), among them, in the 500 mD core, the expanded swept volume is 25.36%, in the 1000 mD core, the expanded swept volume is 27.03%, and in the 2000 mD core, the expanded swept volume is 25.06%.

The results of NMR *T_2_* spectrum curves show that in three different permeability core displacement experiments, polymers with a molecular weight of 16 million and 19 million failed to effectively stay in the water flooding dominant channel of the core, and the subsequent injection water still flows from the channel through which the polymer solution passes. Therefore, subsequent water flooding failed to further expand the swept volume on the basis of polymer flooding. The polymer with a molecular weight of 25 million can stay in the dominant channel of the core for a long time in three different permeability cores, controlling the water flooding mobility, so that the subsequent water flooding can enter the pores that the polymer solution cannot enter, thereby greatly expanding sweep volume.

### 3.3. Change Characteristics of NMR Layering Signals and Imaging in Core Displacement Experiments

**Figure 12:**
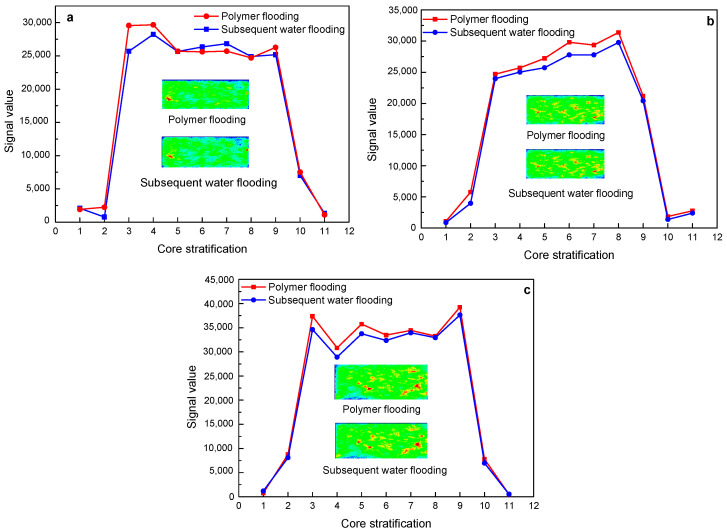
The change characteristics of NMR layering signal and imaging of 16 million molecular weight polymer solutions in rock cores with different permeability after polymer flooding and subsequent water flooding: (**a**) 500 mD core; (**b**) 1000 mD core; (**c**) 2000 mD core.

Figure 12 shows the changes in the nuclear magnetic resonance layering signal and imaging of a polymer with a molecular weight of 16 million during the flooding of three different permeability cores. In the three different permeability core flooding experiments, the subsequent water flooding and polymer flooding have basically the same nuclear magnetic resonance signal values of each layer, and the fluid distribution on the imaging is also basically the same, which is consistent with the results of the NMR *T_2_* spectrum curve.

**Figure 13:**
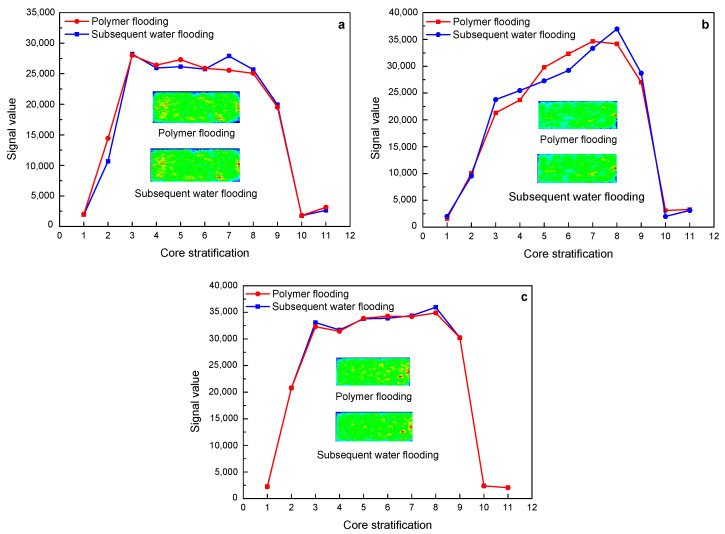
The change characteristics of NMR layering signal and imaging of 19 million molecular weight polymer solutions in rock cores with different permeability after polymer flooding and subsequent water flooding: (**a**) 500 mD core; (**b**) 1000 mD core; (**c**) 2000 mD core.

Figure 13 shows the changes in the nuclear magnetic resonance layering signal and imaging of the polymer with a molecular weight of 19 million during the core flooding process of three different permeability. Similar to the polymer solution with a molecular weight of 16 million, the subsequent water flooding and polymer flooding have basically the same signal values of each layer of nuclear magnetic resonance, and the fluid distribution on imaging is also basically the same, which is consistent with the results of the NMR *T_2_* spectrum curve.

**Figure 14:**
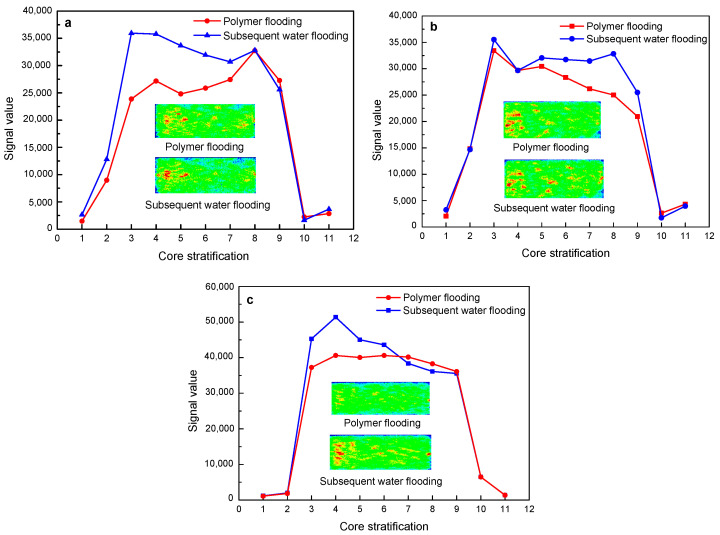
The change characteristics of NMR layering signal and imaging of 25 million molecular weight polymer solutions in rock cores with different permeability after polymer flooding and subsequent water flooding: (**a**) 500 mD core; (**b**) 1000 mD core; (**c**) 2000 mD core.

Figure 14 shows the changes in NMR layering signals and imaging of the polymer solution with a molecular weight of 25 million during the flooding of three different permeability cores. As shown by the nuclear magnetic resonance layered signal value curves, in the three sets of core displacement experiments, the signal value of subsequent water flooding increases to varying degrees on the basis of polymer flooding. In the 500 mD and 2000 mD cores, the subsequent water flooding mainly increases the signal value of the front end of the core on the basis of polymer flooding. It can also be seen from the imaging that the color of the fluid at the front end of the subsequent water flooding core is darker than that of the polymer flooding, indicating an increase in the signal value. In the 1000 mD core, the subsequent water flooding mainly increases the signal value at the back end of the core on the basis of polymer flooding.

Through the injection performance experiments of polymer solutions with different molecular weights in rock cores with different permeability, and analysis from four aspects (injection pressure, NMR *T_2_* spectrum, NMR layered signal, and imaging), it is possible to more accurately determine the matching adaptability between the molecular weight of the polymer and the permeability of the rock core.

## 4. Conclusions

Using nuclear magnetic resonance technology, through the injection core experiment of polymer solution, the change characteristics of four aspects of injection pressure, nuclear magnetic resonance *T_2_* spectrum, nuclear magnetic resonance layering signal, and imaging are analyzed, which can accurately characterize the adaptive matching relationship between polymer molecular weight and rock core permeability.

As the molecular weight increases, the injection pressure of the three different permeability cores increases accordingly. It is difficult for the polymer solutions with a molecular weight of 16 million and 19 million to stay in the core for a long time, and the subsequent water flooding pressure decreases rapidly. The polymer with a molecular weight of 25 million can stay in the core for a long time and control the water flooding mobility.

Among the core flooding experiments of polymers with different molecular weights, the polymer with a molecular weight of 25 million performed the best. In the 500–2000 mD core, the dominant channel of water flooding can be well controlled, and the subsequent water flooding can further enter the pores that the polymer solution cannot reach. Subsequent water flooding can expand the swept volume by about 25% compared with polymer flooding, thereby greatly improving oil recovery.

## Figures and Tables

**Table 1:** Core data.

Core Number	Diameter × Length/cm	Permeability/mD	Porosity/%
500-1	2.51 × 4.94	519	26.4
500-2	2.51 × 4.92	519	26.3
500-3	2.51 × 4.89	512	26.5
1000-1	2.51 × 4.76	1036	26.8
1000-2	2.51 × 4.93	983	27.0
1000-3	2.51 × 4.98	983	27.3
2000-1	2.51 × 5.13	2058	32.1
2000-2	2.51 × 4.88	2076	32.3
2000-3	2.51 × 5.24	2076	37.3

## Data Availability

Data availability available based on request.
